# miR-23b Negatively Regulates Sepsis-Induced Inflammatory Responses by Targeting ADAM10 in Human THP-1 Monocytes

**DOI:** 10.1155/2019/5306541

**Published:** 2019-10-31

**Authors:** Wenying Zhang, Furong Lu, Yuliu Xie, Yao Lin, Tian Zhao, Shoubao Tao, Zhipeng Lai, Ning Wei, Ruoxuan Yang, Yiming Shao, Junbing He

**Affiliations:** ^1^The Intensive Care Unit, Affiliated Hospital of Guangdong Medical University, Zhanjiang, Guangdong, China; ^2^The Intensive Care Unit, Jieyang Affiliated Hospital, Sun Yat-sen University, Jieyang, Guangdong, China

## Abstract

**Background:**

Previous studies have demonstrated pivotal roles of disintegrin and metalloproteinase 10 (ADAM10) in the pathogenesis of sepsis. MicroRNA- (miR-) 23b has emerged as an anti-inflammatory factor that prevents multiple autoimmune diseases. However, the underlying mechanisms of miR-23b in the regulation of ADAM10 and sepsis remain uncharacterized.

**Methods:**

The expression levels of ADAM10 and miR-23b were detected by quantitative RT-PCR and western blot analysis. Cytokine production and THP-1 cell apoptosis were measured by enzyme-linked immunosorbent and annexin V apoptosis assays. Bioinformatics analyses and qRT-PCR, western blot, and luciferase reporter assays were performed to identify ADAM10 as the target gene of miR-23b.

**Results:**

miR-23b expression was downregulated in the peripheral blood mononuclear cells of sepsis patients and LPS-induced THP-1 cells and was negatively correlated with the expression of ADAM10 and inflammatory cytokines. miR-23b regulated ADAM10 expression by directly binding to the 3′-UTR of ADAM10 mRNA. The overexpression of miR-23b alleviated the LPS-stimulated production of inflammatory cytokines (TNF-*α*, IL-1*β*, and IL-6) and apoptosis by targeting ADAM10 in THP-1 cells. The inhibitor or knockdown of ADAM10 elicited effects similar to those of miR-23b on THP-1 cells upon LPS stimulation.

**Conclusions:**

The present study demonstrated that miR-23b negatively regulated LPS-induced inflammatory responses by targeting ADAM10. The molecular regulatory mechanism of miR-23b in ADAM10 expression and sepsis-induced inflammatory consequences may provide potential therapeutic targets for sepsis.

## 1. Introduction

Sepsis is defined as a systemic inflammatory reaction syndrome resulting from infection by various pathogenic microorganisms; it results in distressingly high morbidity and mortality in intensive care units (ICUs) throughout the world [1, 2]. Despite the unclear pathological mechanisms of sepsis, the progression of inflammatory cascades and the release of various proinflammatory mediators have been demonstrated to be involved in the pathogenesis and development of sepsis [3, 4]. A disintegrin and metalloproteinase 10 (ADAM10) is involved in the shedding of more than 40 cellular substrates, including CX3CL1, IL-6R, and TNF-*α*, and influences the release of the inflammatory cytokines IL-6 and IL-1*β* in activated neutrophils, monocytes, and macrophages, which have been theorized to play pivotal roles in sepsis [5–7]. ADAM10 expression levels were significantly increased in septic patients and animal models of sepsis and were markedly associated with disease severity and mortality [7, 8]. In addition, the specific antagonist or genomic deletion of ADAM10 contributes to a significant decrease in septic response and endothelial barrier disruption and confers a protective benefit against sepsis to mice [9, 10]. These lines of evidence indicate that the blockade of ADAM10 may be a promising therapeutic target for sepsis.

MicroRNAs (miRNAs), a class of endogenous, single-stranded and noncoding small RNAs, can posttranscriptionally regulate gene expression by degrading mRNAs and inhibiting their translation by targeting the 3′-untranslated region (3′-UTR) of mRNAs, which play pivotal roles in the regulation of immune cell activation, inflammatory cytokine release, and immune response [11–13]. Previous studies have shown that significant changes in miRNA expression, such as miR-23b [14], miR-146a [15], miR-150 [16], and miR-223 [17], are associated with sepsis. miR-23b, a multifunctional miRNA, has emerged as an anti-inflammatory factor that contributes to the modulation of multiple signaling pathways, in which the molecules regulate dozens of proinflammatory cytokines [18, 19]. Zhu et al. showed a significant decrease in miR-23b expression in patients with rheumatoid arthritis as well as in animal models of multiple sclerosis and rheumatoid arthritis, and miR-23b prevented various inflammatory autoimmune diseases by targeting IL-17 [20]. Other evidence has indicated that miR-23b plays crucial roles in the pathomechanism of sepsis by suppressing the production of inflammatory cytokines, including NF-*κ*B, TNF-*α*, IL-6, IL-1*β*, and E-selectin [21]. Nonetheless, the detailed biological function and the potential regulatory mechanisms of miR-23b in the inflammatory response following sepsis have not been completely elucidated.

Bioinformatics analyses showed that a highly conserved putative binding site existed between the 3′-UTR of the ADAM10 mRNA and miR-23b. However, the specific role of miR-23b in the regulation of ADAM10 and sepsis remains unclear. Given the evidence suggesting that ADAM10 and miR-23b play pivotal roles in the pathogenesis of sepsis, we carried out this study to evaluate the association between ADAM10, miR-23b, and sepsis. Moreover, in vitro functional experiments were also conducted to identify the role of miR-23b in regulating inflammatory responses by targeting ADAM10 following sepsis. The underlying mechanism of miR-23b in the regulation of ADAM10 expression and sepsis-induced inflammatory responses may provide new opportunities for the development of potential therapeutic interventions against the disease.

## 2. Methods

### 2.1. Subject Enrollment

In this study, a total of 30 patients with sepsis (mean age: 59.77 ± 2.79 years; 63.3% male) admitted to the intensive care unit (ICU) at the Affiliated Hospital of Guangdong Medical University were enrolled between September 2016 and March 2017. All patients met the International Sepsis Definitions Conference diagnostic criteria for sepsis (2012), without suffering from diabetes mellitus, cancer, autoimmune diseases, and human immunodeficiency virus or receiving immunosuppressive, steroid, or radiation therapy [22]. During the same period of time, 30 healthy subjects (mean age: 53.67 ± 2.01 years; 70.0% male) without cancer, autoimmune diseases, sepsis, and other inflammatory diseases from the Health Examination Center in this hospital were recruited as the control group. The blood specimens were collected in the 12 h period when the diagnosis of sepsis was established. Written informed consent from the studied subjects or their families was obtained prior to their enrollment. This study was approved by the Affiliated Hospital of Guangdong Medical University Ethical Committee.

### 2.2. Plasma Collection and Mononuclear Cell Isolation

Serum was separated from the blood specimens of all participants by centrifugation at a speed of 400 x g for 15 minutes at 4°C and stored at -80°C. To isolate the peripheral blood mononuclear cells (PBMCs), the blood specimens were combined with an equal volume of 0.9% NaCl and layered above a Ficoll-Paque PREMIUM solution. After centrifugation at a speed of 800 x g for 30 minutes, the PBMCs formed a distinct band at the media interface. Subsequently, we transferred the cells from the upper white layer to another centrifuge tube, and then an equal volume of 0.9% NaCl was added and mixed gently. Finally, the samples were centrifuged again at a speed of 250 x g for 15 minutes, and the PBMCs that we obtained were resuspended in 1 mL ice-cold 1x PBS to obtain a PBMC precipitate.

### 2.3. Cell Culture, Lentiviral Infection, and LPS Treatment

Human umbilical vein endothelial cells (HUVECs) and THP-1 cells obtained from the American Type Culture Collection (ATCC; Manassas, VA, USA) were cultured in a DMEM and RPMI 1640 medium (Thermo Fisher Scientific) supplemented with 10% fetal bovine serum (Invitrogen) and penicillin/streptomycin (Sigma-Aldrich) at 37°C with 5% CO_2_. The construction of the pGLV3-miR-23b plasmid and lentivirus packaging was designed and synthesized by GenePharma, Inc. (Shanghai, China). The resultant lentivirus containing the miR-23b mimic (LV3-miR-23b mimic), the miR-23b inhibitor (LV3-miR-23b-inhibitor), and the negative control (NC) sequences (LV3-NC) was used to infect HUVECs and THP-1 cells to establish stably overexpressed cell lines. The hsa-miR-23b mimics included 5′-ATCACATTGCCAGGGATTACC-3′, the hsa-miR-23b inhibitor included 5′-GGTAATCCCTGGCAATGTGAT-3′, and the miR-NC included 5′-TTCTCCGAACGTGTCA -CGT-3′. Three interference vectors LV3-shRNA specific to ADAM10 were obtained from Longqian Biotech (Shanghai, China): shRNA1, 5′-GCTGTGCAGATCATTCAGTAT-3′; shRNA2, 5′-CCCTACAAATCCTTTCCGTTT-3′; and shRNA3, 5′-GCAGGTTCTATCTGTGAGAAA-3′. The LV3-NC was set as the negative control that did not target any known genes. These LV3-shRNA vectors were transfected into THP-1 cells using RFect Plasmid DNA Transfection Reagent (BioDai, Changzhou, China). The ADAM10 mRNA expression and protein production were detected by quantitative real-time PCR and western blot to evaluate the transfection efficiency. Cells were stimulated with 500 ng/mL LPS for 12 h. The ADAM10 inhibitor GI254023X (MedChem Express, Princeton, NJ, USA) was dissolved in dimethyl sulfoxide (DMSO) and applied to cells at a concentration of 20 *μ*M with RPMI 1640 supplemented with 10% FBS and 1% antibiotic mixture. Treatment with a control medium was used for control cells.

### 2.4. Quantitative Real-Time PCR (qRT-PCR)

For qRT-PCR analysis of ADAM10 mRNA and miR-23b expression, total RNA was extracted using TRIzol reagent (Sangon Biotech, Shanghai, China) and then converted into cDNA by a First Strand cDNA Synthesis Kit (Thermo Fisher Scientific) according to the manufacturer's instructions. Quantitative RT-PCR was conducted using the SYBR Green RT-PCR Kit (Takara) in a LightCycler 480 sequence detector system (Roche Applied Science, Laval, Quebec, Canada). The qRT-PCR primer sequences are as follows: ADAM10, 5′-CTGGCCAACCTATTTGTGGAA-3′ (forward) and 5′-GACCTTGACTTGGACTGCACTG-3′ (reverse); *β*-actin: 5′-TCCCTGGAGGCGA-AGAGCTACGA-3′ (forward) and 5′-AGCACTGATATGTTGGCGTACAG-3′ (reverse); miR-23b, 5′-GGTGCTCTGGCTGCTTGG-3′ (forward) and 5′-GCCAAGGTCGTGGTTGCG-3′ (reverse); and U6, 5′-CTCGCTTCGGCAGCACA-3′ (forward) and 5′-AACGCTTCACGAATTTGCGT-3′ (reverse). The expression levels of miR-23b and ADAM10 were measured by using the 2^-*ΔΔ*CT^ method with values normalized to the expression of U6 and *β*-actin, respectively [23].

### 2.5. Western Blot Analysis

The total proteins were extracted from the treated cells using the RIPA lysis buffer (Beyotime, Shanghai, China), and the concentrations of protein were measured using a BCA Protein Assay Kit (Thermo Fisher Scientific). After separation by electrophoresis on a SDS-polyacrylamide gel, the proteins were transferred to a polyvinylidene difluoride (PVDF) membrane (Millipore, Bedford, MA, USA). Immunoblotting was performed at 4°C overnight with anti-ADAM10 (ab1997; Abcam, Cambridge, UK) and anti-*β*-actin antibodies (Santa, CA, USA), followed by HRP-linked secondary antibodies. The blots were visualized using an enhanced chemiluminescence (ECL) detection kit (Millipore, Billerica, MA, USA).

### 2.6. Bioinformatics Prediction and Luciferase Reporter Assay

To understand the regulatory mechanism of ADAM10, several different bioinformatics tools, including miRanda (omictools.com/miranda-tool), TargetScan (http://www.targetscan.org), and miRDB (http://www.mirdb.org), were used to predict miRNA. The wild-type or mutant 3′-UTR of ADAM10, which contained the predicted target site for miR-23b or not, was cloned into the pmirGLO luciferase reporter vector (Promega, Madison, WI, USA), named ADAM10-3′UTR-Wt or ADAM10-3′UTR-Mut. The reporter plasmids pmirGLO-ADAM10-3′UTR-Wt and pmirGLO-ADAM10-3′UTR-MUT were designed and constructed by Longqian Biotech (Shanghai, China). The resultant lentivirus containing the miR-23b mimics (LV3-miR-23b mimics) and the NC sequences (LV3-NC) were used to infect HUVECs and THP-1 cells to establish stably overexpressed cell lines. Then, the cells were cotransfected with pmirGLO-ADAM10-3′UTR-Wt and pmirGLO-ADAM10-3′UTR-MUT using Lipofectamine 2000 (Invitrogen, USA) following the manufacturer's instructions. After 24 h of incubation, the cells were lysed to detect the luciferase activity using a dual-luciferase assay kit (Promega, USA) following the manufacturer's protocol.

### 2.7. Enzyme-Linked Immunosorbent Assay (ELISA) for Cytokine Measurements

The expression levels of TNF-*α*, IL-6, and IL-1*β* in the plasma of studied subjects and the culture supernatants of THP-1 cells were measured using specific commercial ELISA kits (Tiangen Biotech) following the manufacturer's protocol. The concentrations of TNF-*α*, IL-6, and IL-1*β* were calculated by detecting the absorbance at 450 nm with a microplate reader (EpochTM).

### 2.8. Annexin V Apoptosis Assay

An ANXA5/Annexin V-FITC Apoptosis Detection Kit (Beyotime Institute of Biotechnology, Shanghai, China) was used to assess the apoptosis of THP-1 cells following the manufacturer's protocol. Briefly, cells were harvested and gently washed with PBS. After resuspending in 195 *μ*L binding buffer, ANXA5-FITC stock solution (5 *μ*L) and propidium iodide (10 *μ*L) were added. Cells were finally observed and analyzed using a fluorescence microscope and FACS after 15 minutes of incubation at 37°C.

### 2.9. Statistical Analyses

All data are presented as the mean ± SD (standard deviation) from at least three independent experiments. Statistical analyses were carried out using SPSS version 19.0 (IBM, NY, USA) and GraphPad Prism 4.0 (GraphPad Software Inc., San Diego, CA, USA). Differences between groups were analyzed by one-way analysis of variance combined with the Student-Newman-Keuls post hoc test or the nonparametric Mann-Whitney *U* test. *P* < 0.05 was considered an indication of a statistically significant difference.

## 3. Results

### 3.1. miR-23b Is Downregulated, and the Expression of ADAM10, TNF-*α*, IL-1*β*, and IL-6 Is Significantly Elevated in Sepsis Patients

**Figure 1:**
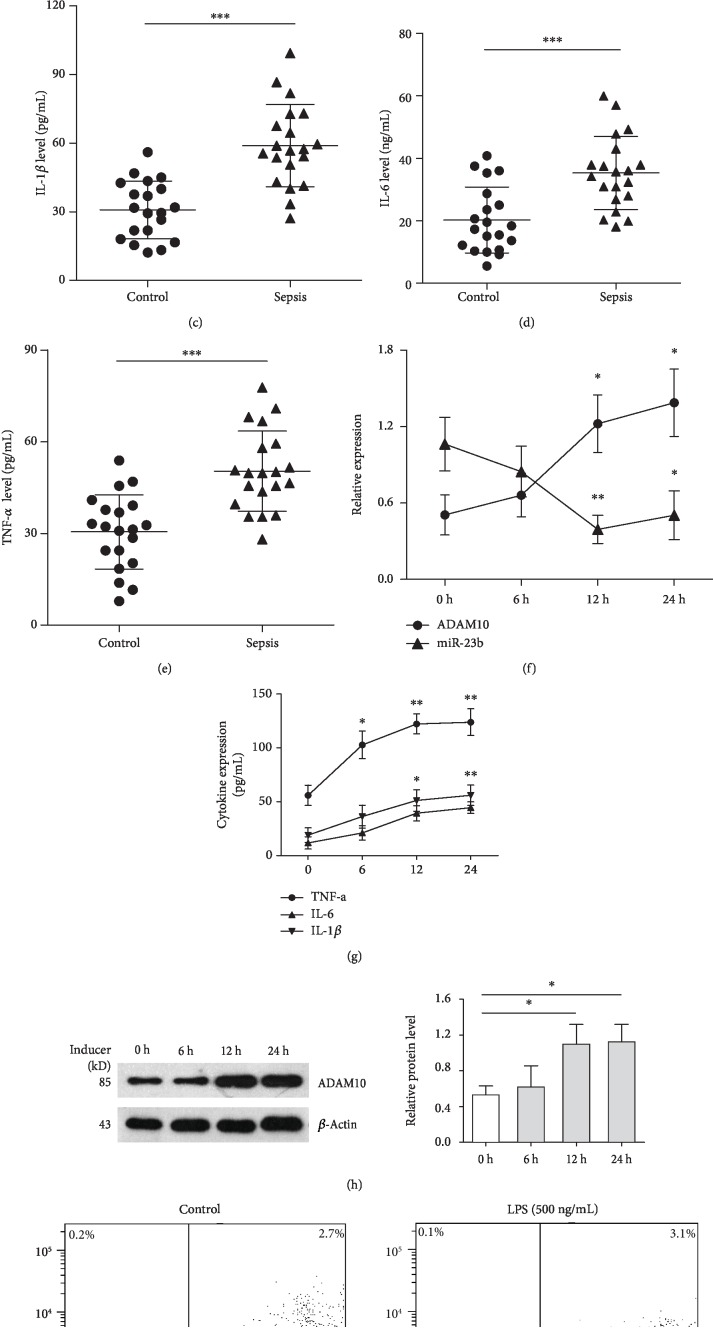
Analysis of miR-23b, ADAM10, inflammatory cytokines, and apoptosis in patients with sepsis and LPS-stimulated THP-1 cells. Peripheral blood mononuclear cells (PBMCs) and serum were extracted from the blood samples of sepsis patients (*n* = 30) and healthy controls (*n* = 30). The expression of miR-23b (a) and ADAM10 (b) in PBMCs was detected by qRT-PCR. The serum concentrations of IL-1*β* (c), IL-6 (d), and TNF-*α* (e) were measured by ELISA. THP-1 cells were treated with LPS (500 ng/mL) for different time periods (6, 12, and 24 hours). miR-23b and ADAM10 mRNA expression was detected by qRT-PCR analysis, and the inflammatory cytokines (IL-1*β*, IL-6, and TNF-*α*) were measured by ELISA (f, g). ADAM10 protein was also measured by western blotting (h). Evaluation of apoptosis of THP-1 cells treated with PBS or LPS (500 ng/mL) for 12 h was performed using flow cytometric analysis of Annexin V-FITC/PI staining (i). Each experiment was independently repeated at least three times. ^∗^*P* < 0.05, ^∗∗^*P* < 0.01, and ^∗∗∗^*P* < 0.001.

In this study, PBMCs and serum were extracted from blood samples of 30 sepsis patients and 30 healthy controls to evaluate the expression of miR-23b and ADAM10 and the inflammatory response in sepsis patients. There was no significant difference in age or gender distributions between the case and control groups (Table 1, all *P* > 0.05). As presented in Figures 1(a) and 1(b), qRT-PCR results showed that the expression of miR-23b was decreased while ADAM10 expression was significantly upregulated in the PBMCs of sepsis patients compared with the healthy controls. Significantly higher serum concentrations of TNF-*α*, IL-6, and IL-1*β* were found in septic patients compared with the controls (Figures 1(c)–1(e)).

### 3.2. LPS Decreases miR-23b Levels, Upregulates ADAM10 Expression, and Induces the Inflammatory Response in THP-1 Cells

We next evaluated whether the administration of LPS regulated miR-23b and ADAM10 expression as well as the inflammatory response. After treatment with LPS (500 ng/mL) for different time periods (6, 12, and 24 h), miR-23b and ADAM10 expression in THP-1 cells were detected by using qRT-PCR and western blot analyses. Flow cytometry and ELISA assays were also performed to detect cell apoptosis and the production of inflammatory cytokines, respectively. Our results showed that LPS significantly increased ADAM10 expression and inflammatory cytokine (TNF-*α*, IL-1*β*, and IL-6) production in a time-dependent manner (Figures 1(f)–1(h)). A significantly high apoptosis rate of THP-1 cells was induced following a 12 h treatment with 500 ng/mL LPS (Figure 1(i)). Conversely, miR-23b expression in cells was significantly downregulated by LPS treatment in a time-dependent manner; it reached the lowest level (decreased by 63%) at 12 h following challenge with 500 ng/mL LPS. These results indicated that miR-23b might be involved in the regulation of ADAM10 expression and the inflammatory response in THP-1 cells upon LPS stimulation.

### 3.3. Overexpression of miR-23b Alleviates LPS-Stimulated Expression of ADAM10 and Inflammatory Response in THP-1 Cells

**Figure 2:**
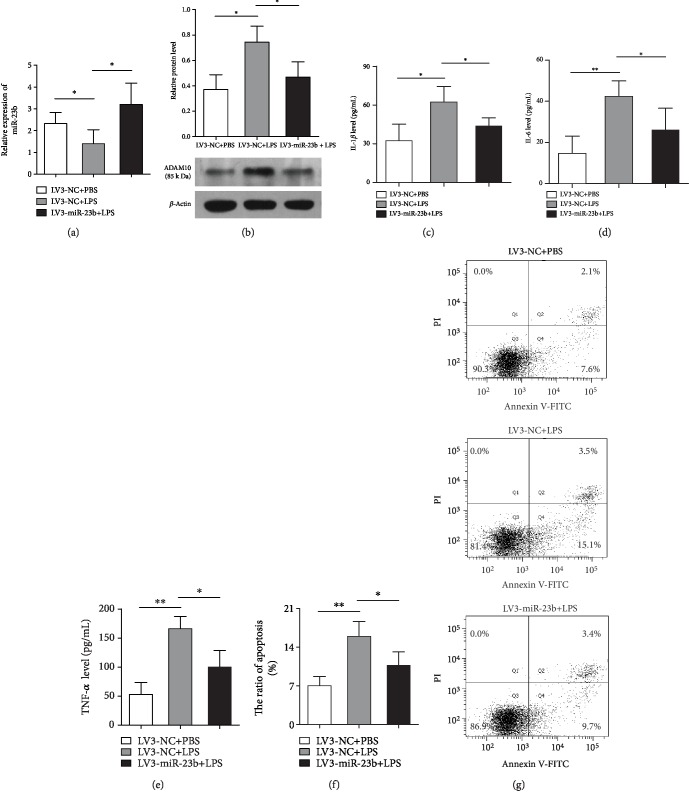
miR-23b alleviates LPS-stimulated ADAM10 expression, inflammatory cytokines production, and apoptosis in THP-1 cells. THP-1 cells were infected by LV3-miR-23b mimics to overexpress miR-23b and then were stimulated by LPS (500 ng/mL) for 12 h. The expression of miR-23b and ADAM10 protein in THP-1 cells was detected by qRT-PCR and western blotting, respectively (a, b). The supernatant concentrations of inflammatory cytokines (IL-1*β*, IL-6, and TNF-*α*) from THP-1 cells were measured by ELISA assay (c–e). Apoptotic cell rates were detected by using flow cytometric analysis of Annexin V-FITC/PI staining (f, g). Each experiment was independently repeated at least three times. ^∗^*P* < 0.05, ^∗∗^*P* < 0.01, and ^∗∗∗^*P* < 0.001.

To evaluate the effect of miR-23b on LPS-stimulated ADAM10 expression and inflammatory response in THP-1 cells, LV3-miR-23b mimics were used to infect THP-1 cells to establish stably overexpressed cell lines prior to the treatment with LPS. As presented in Figure 2(a), qRT-PCR results showed that miR-23b expression in cells infected with LV3-miR-23b was significantly elevated compared with the expression in the NC group upon LPS stimulation for 12 h. The overexpression of miR-23b significantly decreased inflammatory cytokine (TNF-*α*, IL-1*β*, and IL-6) production in THP-1 cells upon LPS stimulation, which indicated an inhibitory effect of miR-23b on the LPS-stimulated inflammatory response (Figures 2(c)–2(e)). LPS treatment induced a striking increase in the apoptotic rate of THP-1 cells, which was significantly attenuated by infection with LV3-miR-23b (Figures 2(f) and 2(g)). These results demonstrated that miR-23b acted as an anti-inflammatory factor in regulating the inflammatory response in sepsis patients. In addition, the LPS-stimulated expression of ADAM10 was observed to be decreased by the overexpression of miR-23b, indicating a regulatory role of miR-23b in ADAM10 expression (Figure 2(b)).

### 3.4. miR-23b Regulates ADAM10 Expression by Directly Targeting the 3′-UTR of ADAM10

**Figure 3:**
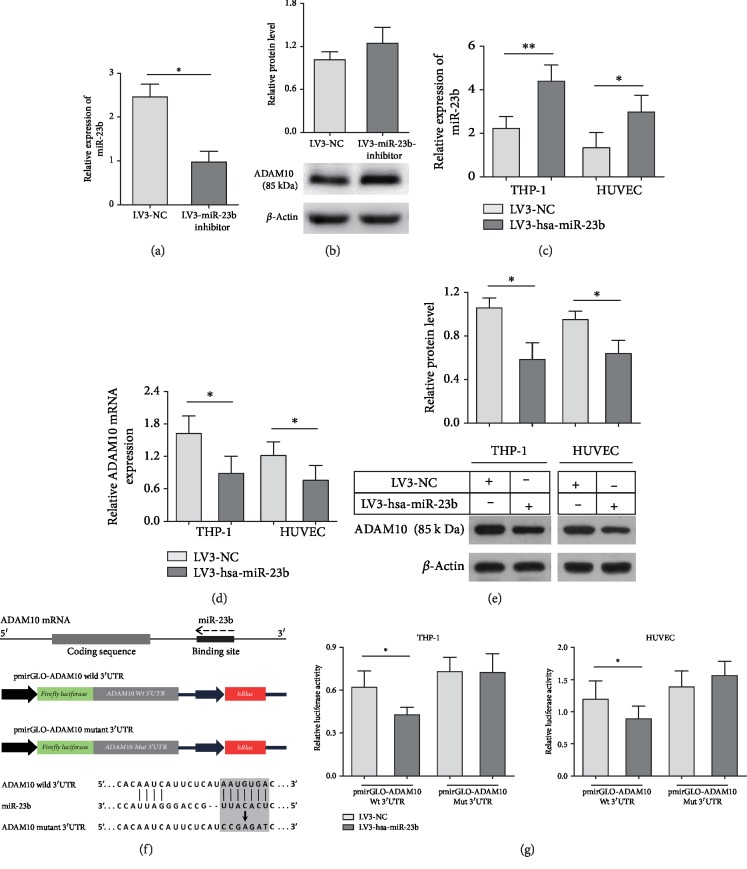
miR-23b regulates ADAM10 expression through directly targeting the 3′-UTR of ADAM10. THP-1 cells were infected by LV3-miR-23b inhibitor or LV3-NC, and the infection efficiency of LV3-miR-23b inhibitor in THP-1 cells was validated using qRT-PCR analysis (a). ADAM10 protein was measured by western blotting (b). HUVECs and THP-1 cells were also infected by LV3-miR-23b mimics or miR-NC. Relative miR-23b and ADAM10 expression was detected by qRT-PCR analysis and western blotting (c–e). Bioinformatics analysis of miR-23b predicted binding sites in the 3′-UTR of ADAM10 and the mutations introduced into the 3′-UTR (f). The luciferase activities of reporter vectors carrying wild-type (Wt) or mutant (Mut) 3′-UTR of ADAM10 were measured in HUVECs and THP-1 cells infected by LV3-miR-23b mimics or LV3-NC (g). Each experiment was independently repeated at least three times. ^∗^*P* < 0.05, ^∗∗^*P* < 0.01, and ^∗∗∗^*P* < 0.001.

Bioinformatics analyses predicted by TargetScan and miRDB showed that a highly conserved putative binding site existed between the 3′-UTR of ADAM10 mRNA and miR-23b, presented in Figure 3. The siRNA-depletion efficiency of the LV3-miR-23b inhibitor in THP-1 cells was validated using qRT-PCR analysis (Figure 3(a)). An increased trend in ADAM10 expression, although not significant, was observed in THP-1 cells with miR-23b knockdown (Figure 3(b)). Next, we infected HUVECs and THP-1 cells with LV3-miR-23b mimics to overexpress miR-23b (Figure 3(c)). qRT-PCR and western blot results showed that ADAM10 expression was significantly downregulated in HUVECs and THP-1 cells infected with LV3-miR-23b mimics compared with the expression in the control group (Figures 3(d) and 3(e)). To validate the specific effect of miR-23b on the regulation of ADAM10 expression by directly targeting the 3′-UTR of ADAM10, the wild-type or mutant 3′-UTR fragments of ADAM10, which included the predicted target site for miR-23b or not, were cloned into firefly luciferase vectors (Figure 3(f)). HUVECs and THP-1 cells were then cotransfected with pmirGLO-ADAM10-3′UTR-Wt or pmirGLO-ADAM10-3′UTR-MUT and LV3-miR-23b mimics or LV3-NC. Our data showed that the overexpression of miR-23b significantly decreased the reporter luciferase activities of the pmirGLO-ADAM10-3′UTR-Wt plasmid, whereas no obvious change was found with pmirGLO-ADAM10-3′UTR-MUT (Figure 3(g)). These results indicated that miR-23b regulated ADAM10 expression through direct binding to the 3′-UTR of ADAM10.

### 3.5. ADAM10 Is Involved in the Regulation of the Inflammatory Response and Apoptosis by miR-23b in THP-1 Cells

**Figure 4:**
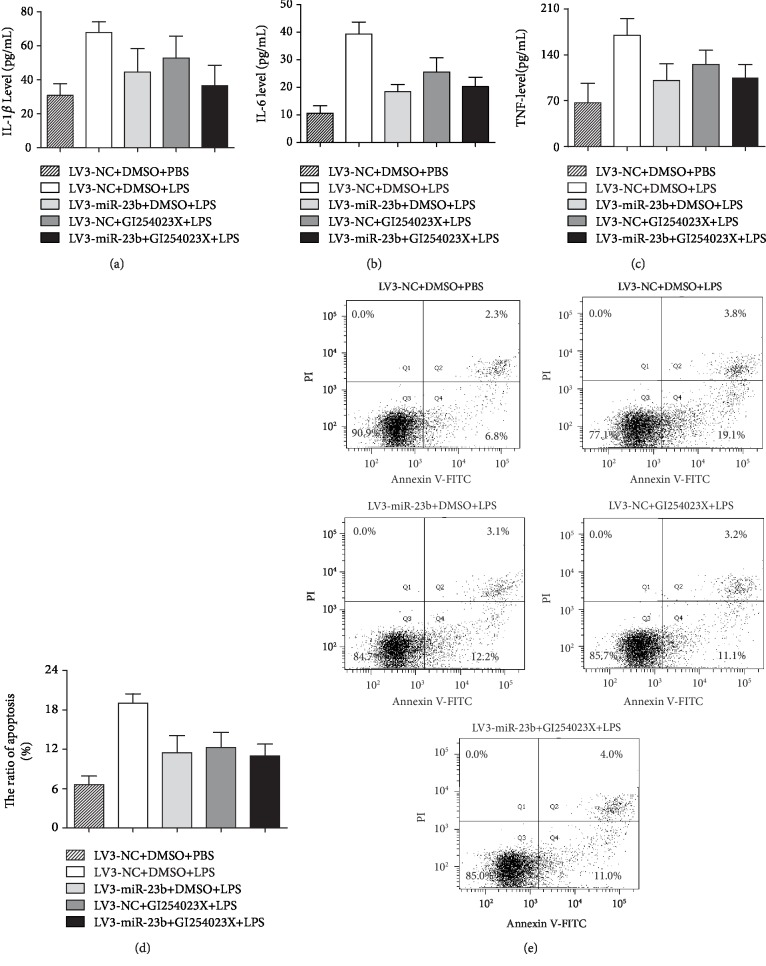
Effects of the interaction between miR-23b overexpression and ADAM10 inhibitor on LPS-stimulated inflammatory response and apoptosis in THP-1 cells. ADAM10 inhibitor GI254023X was applied to THP-1 cells at the concentration of 20 *μ*M. THP-1 cells were infected by LV3-miR-23b mimics to overexpress miR-23b. Either LV3-miR-23b infection or ADAM10 inhibitor significantly decreased the production of IL-1*β*, IL-6, and TNF-*α* and apoptosis in THP-1 cells compared to the control upon LPS stimulation (500 ng/mL), while together did not significantly alleviate the inflammatory responses and apoptosis compared with LV3-miR-23b+DMSO and LV3-NC+GI254023X. Each experiment was independently repeated at least three times.

Next, we suppressed ADAM10 using the specific inhibitor GI254023X to evaluate the effect of ADAM10 on the inflammatory response and apoptosis following LPS treatment. As presented in Figure 4, the inhibitor of ADAM10 significantly decreased the production of TNF-*α*, IL-1*β*, and IL-6, as well as the apoptosis rate in THP-1 cells upon LPS stimulation, which was consistent with that induced by LV3-miR-23b. Furthermore, the cells were incubated with an ADAM10 inhibitor after infection with LV3-miR-23b to validate whether miR-23b negatively regulated LPS-induced inflammatory responses by targeting ADAM10 in human THP-1 monocytes. Our results showed that the combination of LV3-miR-23b infection and GI254023X treatment did not significantly decrease the LPS-stimulated production of TNF-*α*, IL-1*β*, and IL-6 and the apoptosis rate in THP-1 cells compared with LV3-miR-23b+DMSO and LV3-NC+GI254023X (Figures 4(a)–4(e)).

**Figure 5:**
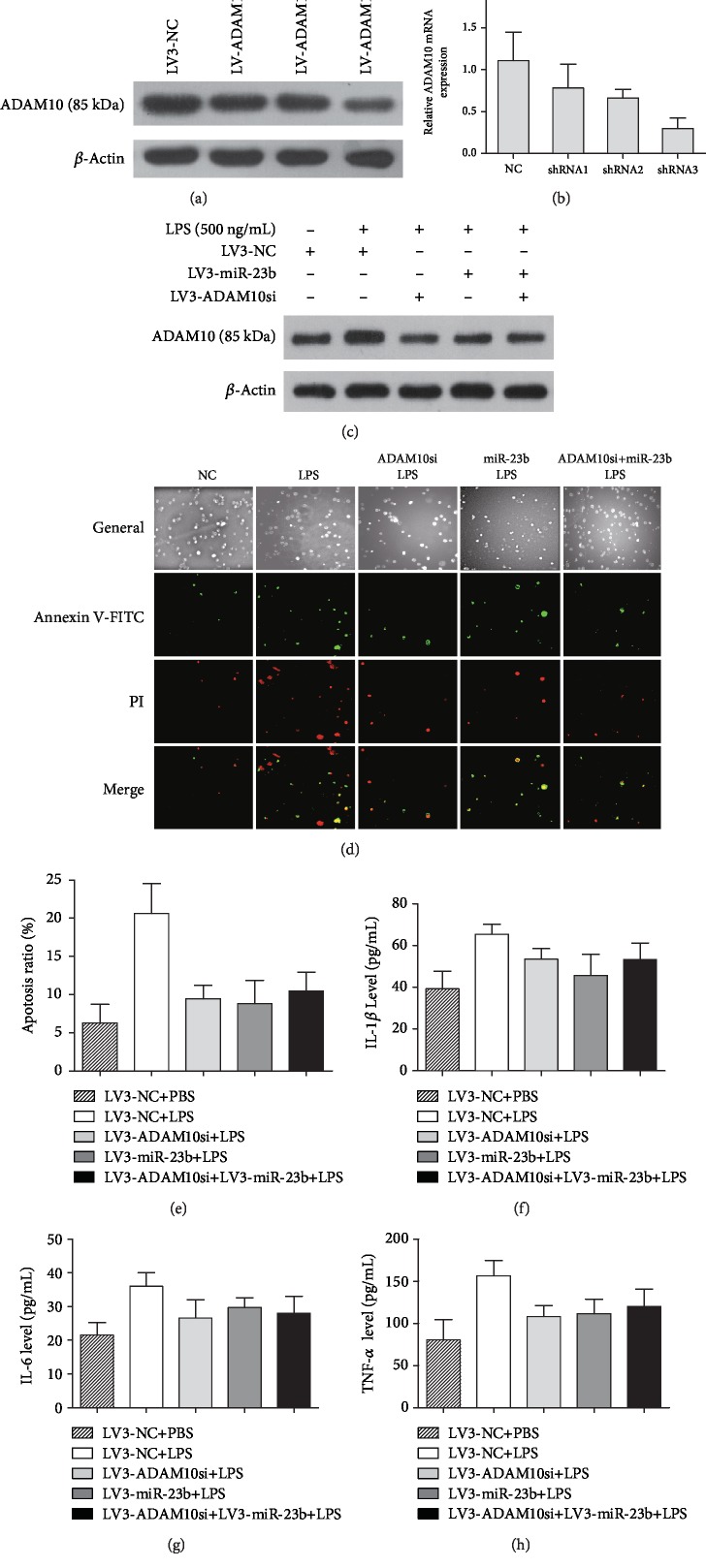
miR-23b alleviates the inflammatory response and apoptosis in THP-1 cells upon LPS stimulation by directly targeting ADAM10. The transfection efficiency of LV3-ADAM10-shRNA in THP-1 cells was evaluated by qRT-PCR and western blot (a, b). The miR-23b mimics and/or ADAM10 knockdown significantly decreased ADAM10 protein production (c). The apoptosis of THP-1 cells detected by the Annexin V-FITC/PI (green/red) double staining assay. The percentage of cells in early and late stages of apoptosis was obtained by analysis of the cell images (mean ± SD, at least 3 experiments) (d, e). Knockdown of ADAM10 significantly decreased the LPS-stimulated production of inflammatory cytokines (IL-1*β*, IL-6, and TNF-*α*) and apoptosis rate in THP-1 cells (e–h). Combination of miR-23b mimics and ADAM10 knockdown did not significantly decrease the LPS-stimulated inflammatory response and apoptosis of THP-1 cells compared with miR-23b mimics or ADAM10 knockdown.

We further evaluated the effect of ADAM10 knockdown on LPS-stimulated production of inflammatory cytokines and apoptosis. As shown in Figures 5(a) and 5(b), transfection with LV3-ADAM10-shRNA3 in THP-1 cells led to an effective knockdown of ADAM10 mRNA and protein expression, which was selected to silence the ADAM10 expression in the following experiments. The miR-23b mimics and/or ADAM10 knockdown significantly decreased ADAM10 protein production (Figure 5(c)). Knockdown of ADAM10 significantly decreased the LPS-stimulated production of inflammatory cytokines (IL-1*β*, IL-6, and TNF-*α*) and apoptosis rate in THP-1 cells (Figures 5(d)–5(h)). These results were similar to the phenotype of cytokine production and apoptosis induced by miR-23b. However, combination of miR-23b overexpression and ADAM10 knockdown did not further reduce the production of inflammatory cytokines (IL-1*β*, IL-6, and TNF-*α*) and apoptosis rate in THP-1 cells upon LPS stimulation.

## 4. Discussion

Sepsis is a systemic inflammatory disease resulting from a detrimental reaction to infection by pathogenic microorganisms. It has been demonstrated that the disruption of immune and inflammatory responses plays a key role in the pathogenesis of sepsis [3, 4]. Human immune cells are activated by pathogenic microorganisms and toxins and produce various inflammatory cytokines, leading to serious cell damage, microcirculatory disturbance, and organ dysfunction [24, 25]. As pivotal posttranscriptional regulators of gene expression, miRNAs play a pivotal role in modulating the immune system and inflammatory response during sepsis [26, 27]. In this study, we show for the first time that miR-23b regulates ADAM10 expression by directly targeting the 3′-UTR of ADAM10. Furthermore, miR-23b inhibits inflammatory cytokine (TNF-*α*, IL-1*β*, and IL-6) production and apoptosis in THP-1 cells through ADAM10 upon LPS stimulation. Our findings may advance our understanding of the role of miR-23b and ADAM10 in modulating the biological processes of inflammatory responses in sepsis.

Several lines of evidence have demonstrated that ADAM10 plays significant roles in the pathological mechanism of sepsis [7–10]. ADAM10 is a member of the ADAM family, and it serves as an important molecule for the shedding of multiple substrates, including CX3CL1, IL-6R, and TNF-*α*, which regulate various cellular processes involved in excessive inflammatory responses [28–30]. The enhanced expression of ADAM10 in septic mice and patients with sepsis was observed to be closely correlated with disease severity and mortality [7, 8, 31]. In this study, our results indicated an increase in ADAM10 expression in sepsis patients and THP-1 cells upon LPS treatment. The inhibitor and knockdown of ADAM10 significantly decreased the LPS-stimulated production of inflammatory cytokines (IL-1*β*, IL-6, and TNF-*α*) and apoptosis rate in THP-1 cells, which corroborated several previous studies. Powers et al. identified ADAM10 as the receptor for Staphylococcus aureus *α*-hemolysin, and vascular endothelial dysfunction and concomitant inflammatory responses in a murine sepsis model can be attenuated by ADAM10 inhibition [8, 9]. Other studies demonstrated that ADAM10 siRNA and inhibitor exerted protective functions, such as decreasing vascular permeability, alleviating the inflammatory response, and increasing apoptosis resistance [32–34]. This evidence indicates that ADAM10 may serve as an indicator of sepsis severity and that the blockade of ADAM10 may be a promising therapeutic target for sepsis.

miR-23b is one member of the miR-23b/27b/24-1 family cluster encoded by the chromosomal region 9q22.32, which serves as a pleiotropic regulator in various physiological and pathological processes [35]. Recent studies have focused on the role of miR-23b in sepsis. Wu et al. demonstrated that miR-23b regulated sepsis by suppressing the production of several inflammatory cytokines, such as NF-*κ*B, E-selectin, IL-6, and TNF-*α*, in LPS-stimulated endothelial cells [21]. Other studies indicated that the expression of miR-23b was downregulated by LPS, while its overexpression alleviated LPS-stimulated inflammatory injury in ATDC5 cells [36], and it mediated immunosuppression during late sepsis by suppressing the noncanonical NF-*κ*B pathway [37]. In the present study, decreased expression of miR-23b was observed in the PBMCs of sepsis patients and LPS-induced THP-1 cells, which was negatively correlated with the expression of ADAM10 and inflammatory cytokines. Furthermore, the overexpression of miR-23b alleviates the LPS-stimulated production of ADAM10 and inflammatory cytokines (TNF-*α*, IL-1*β*, and IL-6) and apoptosis in THP-1 cells. These results provide further evidence that miR-23b plays an anti-inflammatory role in sepsis, which may be associated with ADAM10 expression.

It has been demonstrated that miRNAs exert biological roles by acting as posttranscriptional regulators through interactions with sites of imperfect complementarity in the 3′-UTR of their target genes [38]. Individual miRNAs can regulate multiple target genes; several miR-23b target genes, such as autophagy-related gene 12 (ATG12), chemokine ligand 7 (CCL7), and E-cadherin, have been identified and validated and play significant roles in inflammatory and cancer-related pathways [39–41]. Bioinformatics analyses revealed a highly conserved putative binding site between miR-23b and the 3′-UTR of ADAM10 mRNA. In this study, we determined whether ADAM10 served as a direct target gene of miR-23b in HUVECs and THP-1 cells. Our results suggest that miR-23b regulates ADAM10, as evidenced by the downregulation of ADAM10 expression induced by miR-23b in HUVECs and THP-1 cells. Furthermore, miR-23b significantly decreased the reporter luciferase activities of pmirGLO-ADAM10-3′UTR-Wt by targeting the 3′-UTR of ADAM10 mRNA, whereas no obvious change was observed in pmirGLO-ADAM10-3′UTR-MUT, which indicated that miR-23b inhibited ADAM10 expression by targeting the 3′-UTR of ADAM10. In addition, miR-23b overexpression or ADAM10 inhibitor significantly decreased the LPS-stimulated production of TNF-*α*, IL-1*β*, and IL-6 and the apoptosis rate in THP-1 cells and together did not further alleviate the inflammatory responses and apoptosis. These results were similar to the phenotype of cytokine production and apoptosis induced by combination of miR-23b overexpression and ADAM10 knockdown. Hence, ADAM10 could act as a downstream mediator of miR-23b involved in the regulation of sepsis-induced inflammatory responses and apoptosis in human THP-1 monocytes.

## 5. Conclusions

In summary, the present study indicates that miR-23b and ADAM10 function as negative regulators in LPS-induced inflammatory cytokine production in THP-1 monocytes. Bioinformatics analyses and functional in vitro assays demonstrated that miR-23b regulated ADAM10 expression and suppressed inflammatory responses by direct binding to the 3′-UTR of ADAM10 upon LPS stimulation. The molecular regulatory mechanism of miR-23b in ADAM10 expression and sepsis-induced inflammatory consequences may provide new opportunities for the development of potential therapeutic interventions against the disease.

## Figures and Tables

**Table 1:** Demographic characteristics of sepsis patients and healthy controls.

Variable	Sepsis (*n* = 30)	Control (*n* = 30)	*P* value
Age, years, mean ± SEM	59.77 ± 2.79	53.67 ± 2.01	0.081
Male/female, number	19/11	21/9	0.584

## Data Availability

All data generated or analyzed during this study are included in this published article.
